# Creating Digital Sci-Fi Narratives through Multimodal Composing to Promote Adolescent Students’ STEM Education

**DOI:** 10.1186/s43031-023-00072-7

**Published:** 2023-05-12

**Authors:** Hua Ran, Ji Shen, Blaine E Smith, Changzhao Wang

**Affiliations:** 1 Department of Mathematical Sciences, University of Delaware, Ewing Hall, Newark, DE 19716 USA; 2 Department of Teaching and Learning, University of Miami, Coral Gables, FL USA; 3 Department of Teaching and Learning, Peabody College of Education, Vanderbilt University, Nashville, TN USA

**Keywords:** Multimodal composition, Science integration, Science fiction

## Abstract

Much research has examined the incorporation of academic and scientific writing in science learning. However, less research has applied a narrative approach that represents events in a time sequence. Furthermore, modern technology has greatly extended students’ modes of science expression beyond the printed texts. Yet, connecting students’ rich experience in digital storytelling with their academic learning in science is still an area of needed research. This study focused on analyzing the products students generated as a way to examine how they integrated science in their multimodal sci-fi narratives. These narratives were created in a program designed to engage adolescents in integrated STEM and digital literacy learning. More specifically, this study developed a two-dimensional framework (science and integration) to evaluate the 35 products produced by 136 participants in 5 iterations of the program. Content and thematic analyses revealed that a wide variety of sophisticated mechanisms was applied for science integration, including: (1) building connections among diverse science topics; (2) leveraging innovative narrative techniques; (3) responding critically to socio-scientific problems; and (4) designing and redesigning multimodal elements.

**Supplementary Information:**

The online version contains supplementary material available at 10.1186/s43031-023-00072-7.

## Introduction

Writing has been viewed as a promising means for students to shape their understanding of science and communicate their ideas with others (Hand, 2017; Prain & Hand, 2006; Santa & Havens, 1991; Yore, 2000). However, there are mixed perspectives on what genres of writing are best suited to assist students’ science learning. Many scholars argue that writing for science learning should mainly focus on academic and scientific writing, including laboratory or research reports (Halliday & Martin, 1993), argumentative or persuasive writing (Chen et al., 2013), and expository essays (Balgopal & Wallace, 2013). These genres typically employ formal structure and scientific language. However, others suggest that these academic genres may discourage students from leveraging their own vocabularies and connecting with personal experience (Hildebrand, 2004; McDermott & Hand, 2010; Yore et al., 2003). Instead, many researchers recommend using narrative, given its open-ended and flexible nature (Avraamidou & Osborne, 2009; Millar & Osborne, 1998; Norris et al., 2005; Torrance & Galbraith, 1999). On the one hand, this storytelling approach is found to be effective for students’ science conceptualization (e.g., students’ understanding of physical and chemical change, Demircioğlu et al., 2013). What is more, narrative has been considered as more compatible with a constructivist approach because it can be used to connect to learners’ personal experiences and promote deep meaning-making activities (Anilan, 2018; Levinson, 2008; McKee & Ogle, 2005; Mott et al., 1999). As a result, this genre has been gradually used in learning about diverse science topics, including biosecurity (Ritchie et al., 2011), energy resources (Castek & Dwyer, 2018), and climate changes (McKnight, 2010; Tanner, 2010).

Fast development in information and communication technology affords educators the opportunity to use narratives in new ways, such as digital storytelling (Sadik, 2008; Yang, 2012), transmedia storytelling (Jenkins et al., 2018), video blogging or digital graphic novels (Crompton et al., 2019), and 3D narrative environment (Mott & Lester, 2006).

These approaches typically use digital modalities other than printed texts. Multimodal narrative composition in this paper refers to the practice of storytelling through the use and coordination of semiotic resources in multimodal forms (e.g., images, photographic, animation, music, sound, gestures, and videos). The ways of organizing these forms in narratives may be significantly different from the ones in the sole mode of printed texts (Kress & Van Leeuwen, 2001; Lambert, 2013; Nilsson, 2008).

While considerable work has been undertaken to understand the benefits of multimodal narrative composition to develop students’ literacy skills (Smith et al., 2021; Belet & Dala, 2010; Lenters & Winters, 2013; Sarıca & Usluel, 2016), much less research has applied this genre in science education. The uniqueness of using multimodal narratives in science is that students’ understanding of science concepts is embedded and grounded in the narratives’ characters, background, settings, and events (Matuk et al., 2019).

However, without a systematical analytical framework to evaluate student-generated multimodal narratives, it is difficult to fully understand how this genre can be useful for students’ science and literacy learning. This study focuses on developing an analytic framework to examine how groups of students express and integrate science ideas through multimodal composition. The study is situated in Project STEM + L, a program designed to engage adolescent students (grade 5th-8th) in STEM learning through producing multimodal sci-fi narratives. This project creates unique opportunities for studying the use of multimodal narrative composition for engaging students in science learning activities. The combination of multimodal composition with the requirement of embedding science contents makes it challenging, but also affords opportunities, at the same time, to assess student-generated products. This study was especially interested in examining the unique approaches that students may employ in their multimodal sci-fi narratives to integrate science elements. The guiding question for this study is in what ways adolescent students can integrate science elements in their multimodal sci-fi narratives.

## Literature Review

### Using sci-fi for (Environmental) Science Education

Science fiction (sci-fi) is a broad genre of speculative fiction that deals with the advancement of issues related to science and technology, such as time and space travel, extraterrestrial life, alternative universe, and futuristic utopias (Roberts, 2002). Sci-fi has been used in science classrooms in order to enhance learning by drawing on students’ interest and enthusiasm (Kosky, 2014; Vrasidas et al., 2015; Zhang & Callaghan, 2014).

There has been a long tradition of using sci-fi in environmental science education. One reason is that sci-fi may stimulate public discussion and reflection on scientific and technological development and their impacts on human society and environments (Bina et al., 2017; Guerra, 2009; Petersen et al., 2005; Van Dijck, 1999). Sci-fi directly addresses peoples’ concerns, fears, anxiety, and desire to explore new possibilities. Gough (1993) argues that fictional narratives may be more appropriate for representing science and nature than expository or persuasive texts. This is not only because fictional narratives are typically more engaging, but also because the narrative structure is better suited to explore the concepts and problems related to complex environmental issues.

A priority of environmental science education is preparing youth to address increasingly distressing environmental problems. Czerneda (2006) explains that the “what if?” scenarios in sci-fi, for instance, can engage learners in digging deeply into the socio-scientific issues such as climate change with imagination and creativity. This position resonates with Heinlein’s (1969) perspective that sci-fi through speculative experiments can engage the audience to entertain alterative solutions to tackle real world problems. In the project Climate Change and Me, fictional narrative composition is used to immerse high-school students in responding to the moral, ethical, and scientific complexity of climate change (Rousell et al., 2017). At the elementary level, fourth-grade students are engaged in designing fictional characters in order to creatively and critically think about climate present and futures (Morrison & Chisin, 2017).

Evaluating student-generated sci-fi can be challenging. The problem stems from the fact that scientific knowledge in science fiction usually contradicts with what is widely believed, and students’ imagination increases this difficulty. Research provides some alternative paths to investigate “science” in science fiction. One suggestion from science fiction research is to extend the regular science concepts to broader science topics, such as imaginary science (i.e., time travel and alternative university, antigravity, instantaneous communications), men and supermen (biological engineering), and intelligent machines (Nicholls, 1983). Additionally, science fiction often tackles scientific problems from the real world, then problem-solution analysis can also be a useful method (Gess, 2017).

### Multimodal Composition in (Environmental) Science Education

Research has shown that multimodal narratives have the capacity to motivate students in a different manner than traditional texts, including encouraging students to be innovative and creative in delivering messages (Smith et al., 2021; Ercan, 2014; Lenters & Winters, 2013). Furthermore, multimodal composing offers impactful opportunities for culturally and linguistically diverse students to incorporate their personal experiences and to express their knowledge and interests in innovative ways (Nilsson, 2010; Yang, 2012).

A key concept to understand how learning happens in a multimodal learning environment is *design*, referring to the situated sign-making process in which designers create and arrange multimodal semiotic resources to orchestrate and deliver their intended meaning (Kress, 2003; Kress et al., 2001). During the design process, two actions, transformation and transduction, are placed in the center (Kress, 2003). Transformation involves working on and rearranging the structure or elements within one mode to create different meanings (i.e., changing the size, color, or position of one mode); transduction involves reconfiguring and reshaping across different modes based on their affordances (i.e., representing the printed texts into the form of video or comic). These two processes require learners to be innovative, creative, and active designers to better shape and reshape ways of presenting multimodal messages.

Research in recent years suggests that multimodal composition has the potential for environmental science education. Goulah (2017) points out that creating multimodal narratives provides opportunities for 11th grade immigrant students to foster their transformative perspectives and eco-ethical consciousness about climate issues. Castek and Dwyer (2018) have found that, in a project where elementary students are asked to design multimodal posters on renewable energy sources, they can express their thinking in flexible ways through the integration of drawings, images, and descriptions and use their creativity to pose workable solutions to real-world environmental problems.

### Assessment of Multimodal Composition

The current assessment practices often focus on some common narrative elements to grade students’ multimodal work, such as organization or structure, point of view, focus, dramatic tension, and event development (Murray et al., 2010; Zoetewey & Staggers, 2003). However, Sorapure et al. (2006) reminded that over-highlighting on narrative components may lose the chance to see new values emerging from new media.

Some researchers argue that the criteria for assessing multimodal texts must consider how multimodality serves as a means to convey the message. For example, Odell and Katz (2009) suggest that, in the multimodal texts, the evaluation of student abilities needs to include how students:

(1) integrate given information and multimedia;

(2) select, use, and encode multimodal elements; and.

(3) present a logical relationship among multimodal elements.

Yancey (2004) argues that coherence is a key and defining feature of all effective digital composition, indicating how different modes relate to one another to support the overall argument. In a project where college students engage in the creation of digital video scientific documentary, Hafner and Ho (2020) adopt the coherence criterion: i.e., how a variety of multimedia (i.e., video clips, pictures, graphs, diagrams) effectively and appropriately support the documentary in a coherent way.

## Research Methodology

### Project implementation

The implementation contexts of Project STEM + L since 2015 is presented in Table 1. Over the five iterations, a total of 136 adolescent students participated in the program and produced 35 multimodal sci-fi narratives. This project was offered either as an afterschool program or STEM elective course with varying durations and different groups of participants. The participant students came from local middle schools in a large southeastern city in the United States. To recruit students for the afterschool program, we sent the flyers to the local library and collaborating teachers and schools to make the information public; the selection was based on a first-come, first-served principle. For the STEM elective course, we found a local, public school who was willing to accommodate the schedule and content of the curriculum. For Round 1, there were 17 students (13 males and 4 females) enrolled in the study who were mainly Latinx students, consistent with the demographics of the school; For Round 2, 9 students (7 males and 2 females) enrolled in the program from different grades (2 fifth graders, 2 sixth graders, 4 seventh graders, and 1 eighth grader); For Rounds 3 and 5, there were 68 students (34 females and 34 males) participated in this study, where the majority was Latinx, consistent with the race/ethnicity distribution at the school level; For Round 4, a total of 42 students participated in the study (26 male and 16 female; 19 Hispanic/Latinx; 14 Black; 4 White; 5 other; 6 fifth graders, 15 sixth graders, 8 seventh graders, and 13 eighth graders).

**Table 1:** Summary information of Project STEM + L

Program	Year	Duration of Program	Number of participants	Grades	Number of final narratives
Afterschool program	Spring 2015	Ten-day afterschool program in a middle school from Jan 15 to Mar 19 (1 h for each)	17	6th -8th	3
Afterschool program	Fall 2015	Ten-day weekend program on a university campus from Sep 12 to Nov 21 (2.5 h each)	9	6th -9th	3
STEM course	Fall 2016	18-week elective STEM course in a middle school (two 2-hour sessions each week)	32	6th	8
Afterschool program	Summer & Fall 2017	One-week summer camp and six face-to-face fall sessions	42	5th -8th	12
STEM course	Fall 2017	18-week elective STEM course in a middle school (two, 2-hour for each week)	36	6th	9

In the project, students engaged in a series of learning activities and benchmark lessons designed to improve their knowledge and skills in technology, science, and writing. The technological tutorials introduced students to a variety of multimodal composition tools that they could use to produce their multimodal narratives. These tools included Scratch (a visual programming environment for creating animations and games, especially for novice programmers, Resnick et al., 2009), MovieMaker or iMovie (a video creation and editing tool users could use to mix video footage with voiceover, text, music, and transitions), and Pixton (an online platform used to create and share comic strips). The students used iKOS, a web-based knowledge organization platform, to construct, share, and organize their narratives (Jiang et al., 2020). The platform supported three distinctive modes of knowledge representation: The *Wiki* mode allowed users to create knowledge entries similar to the popular site Wikipedia.org; The *PicTag* mode allowed learners to tag and annotate pictures or photos; The ConceptMap mode allowed learners to construct concept maps to visualize the connections among scientific concepts as well as among characters or story plots for their narratives. In addition to these modes, iKOS supported the creation of interactive flipbooks. This feature allowed students to publish their multimodal pages and interactive flipbooks both in class and in the public domain.

The science-oriented lessons provided students science learning opportunities through lectures, hands-on activities (e.g., using recyclable materials to design and construct a ‘cool’ house that maintained a low temperature when exposed in the sunlight), online science curriculum (e.g., wise.berkeley.edu), fieldtrips, and invited guest speakers. These lessons focused on different environmental issues. Writing lessons focused on foundational elements to storytelling, how to write dialogues, and ways to integrate different modes for meaning making.

Besides these lessons, students had ample opportunities in the program to work on their final projects: A multimodal sci-fi narrative that incorporates imaginative contents and science descriptions using texts, comics, animation, videos, and audios (Smith et al., 2018). They worked in small groups of 3–5, and each student took one specific role (e.g., designer, scientist, writer) to lead on a specific aspect of their final project (Smith et al., 2019).

### Data Resource and Analysis

While our project produced rich student data (e.g., interviews, videos, field note observations, and students’ narrative compositions), this study only focused on analyzing students’ final products in order to understand what approaches the students leveraged to incorporate science in their narratives.

Particular focus was placed on the science integrated through writing and other modalities. Using a grounded theory methodology (Strauss & Corbin, 1994), an inductive and iterative approach was taken to develop a scoring rubric to evaluate the quality of science integration in students’ multimodal narratives. The rubric consisted of two dimensions—science and integration. The science dimension included two aspects:

- Concepts/phenomena: i.e., how a multimodal narrative depicted a natural phenomenon or defined a science concept;
- Problem/solutions: i.e., how a science-related problem was presented, analyzed, and resolved in the narratives.

The integration dimension also included two aspects:

- Integration between narrative and science: i.e., how each sentence that included science contents was integrated and connected with the rest of the narratives;
- Integration between narrative and problem/solution: i.e., how the problem was presented as a part of the plot through the whole narratives from the beginning to the end.

The specific criteria used in assessing the quality of each aspect was formed during the analysis process (Table 2). The formation of the criteria was an iterative process involving constantly classifying, refining, and redefining. For example, to evaluate the levels of science description/explanation, using accurate, clear, and specific language was considered when referring to science concepts/phenomena. More examples and ways of using the criteria to evaluate students’ work are provided in Appendix A.

**Table 2:** Framework for evaluating multimodal sci-fi narratives

Dimensions	Aspects	Analysis unit	Criteria
Science	Concepts/ phenomena	Science concept/ phenomenon	♣ *Accuracy*: The accuracy and precision of the language/terminologies used to depict or define the science concept/phenomenon ♣ *Clarity*: The clarity and comprehensibility of the description or definition about the science concept/phenomenon ♣ *Specificity*: The specificity and details offered to describe the science phenomena/concepts
	Problem/ solutions	Science-related problems/solution	♣ *Completeness*: The completeness and comprehensiveness of the analysis of the problem, including the cause, seriousness, or consequence of the problem(s) or obstacles that needed to overcome for solving the problem(s) ♣ *Plausibility*: The plausibility, feasibility, and possibility of the solution in terms of science underpinning ♣ *Suitability*: The suitability and level of matching the solutions have in relationship to the problem (based on the context or condition in the narrative)
Integration	Integration between science and narrative	Science-related sentence	♣ *Location relevance*: the science information is placed within the narrative in an appropriate location. ♣ *Embedded-format relevance*: The science information is inserted naturally in the narrative through some techniques (i.e., character dialogue, videos of a news report) ♣ *Event relevance*: The science information is closely related to the event before or after (i.e. providing science explanation for events that already occurred or proving implicit/explicit clues for upcoming events).
	Integration between problem and narrative	Science-related problem	♣ *Set-up (the beginning scene)*: The problem was used to set up the first scene of story, including illustrating story background or introducing the main characters and its relationship. ♣ *Confrontation (the middle scene)*: The problem drove the story to confrontation or climax ♣ *Resolution (the ending scene)*: The problem helped built the last scene of story via leading to resolution or reflection for responding the problem mentioned in the first scene

Each aspect for both dimensions was scored between 0 and 3 using the criteria as shown in Table 2. A score for each dimension was then calculated as the sum of the two aspects. Six narratives (17%) were individually coded by at least two independent coders to establish the inter-rater reliability. The weighted Kappa (*Κ* = 0.83) indicated there was an almost perfect agreement between the coders’ judgement based on Cohen’s (1960) criteria. The disagreement among coders was resolved through iterative group discussion, which helped clarify and produce the final rubrics.

In order to better understand how science was integrated into the narratives, a comprehensive content analysis (Cullum-Swan & Manning, 1994) was further conducted. First, the science-related topics and/or phenomena in all relevant texts and other modalities were identified and grouped into the common themes. Any ideas, concepts, problems, or phenomena in the narratives that were related to science were coded, labeled, and classified into the science themes based on commonality. Then, to address the research questions, a combination of inductive and deductive approaches was used to categorize the mechanisms for science integration (Guest et al., 2011). The scoring rubrics were used as an initial guideline to set up the scope and directions for generating the common themes in science integration. Meanwhile, during the scoring processes, any interesting and potential ways of incorporating science were brought up for further discussion. The process involved iterative discussions and refinement for finding the most appropriate labels and cross-cutting themes pertaining to science integration.

## Research results

### Distribution of narratives on quality of Science and Science Integration

The descriptive statistics showed that both aspects of the science dimension (concepts/phenomena, problem-solution) received a mean score close to 1 (*M* = 1.34, *SD* = 1.05; *M* = 1.20, *SD* = 0.79, respectively), indicating that the narratives fell short in the science aspect. For the integration dimension, the aspect of problem integration (*M* = 1.57, *SD* = 1.00) received a mean score lower than science integration (*M* = 1.91, *SD* = 1.31).

**Fig. 1:**
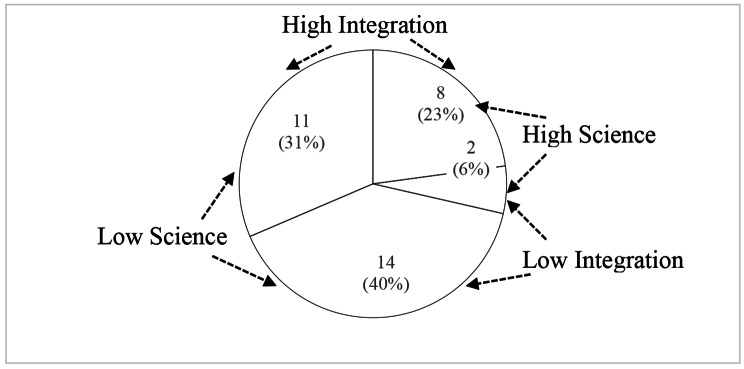
The Distribution of the 35 Narratives based on Quality of Science and Integration

For further analysis of product quality, the two dimensions were split at the middle point to differentiate the work that was high (score > 3.5) and low (score < 3.5). As a result, four profiles (Fig. 1) emerged: (1) Fourteen narratives (14%) were scored low both in the science and integration dimensions, (2) Eleven narratives (31%) were scored high in the integration dimension but low in the science dimension, (3) two narratives (6%) were scored high in the science dimension but low in the integration dimension, and (4) eight narratives (23%) were scored high both in the science and integration dimensions. The distribution indicated that for those narratives that did not present high-quality scientific information (i.e., low science), about half of them (11 out of 25) were able to closely integrate the science in their narratives. In contrast, for those narratives that included better scientific information (i.e., high science), the majority (8 out of 10) presented a high quality in science integration. The different distribution in the two dimensions reflected that students’ narratives had relatively high quality in the integration dimension but low quality in the science dimension.

### Diversity of Science Integration Mechanisms

Further content analysis of students’ products revealed that they applied different mechanisms to integrate science into their narratives. These mechanisms included diversity of science topics and their connections, integrating science information through various narrative techniques, proposing unique solutions for science-related problems, and integrating science using multiple modes.

### Theme1: diversity of Science Topics and cross-topic connections

**Fig. 2:**
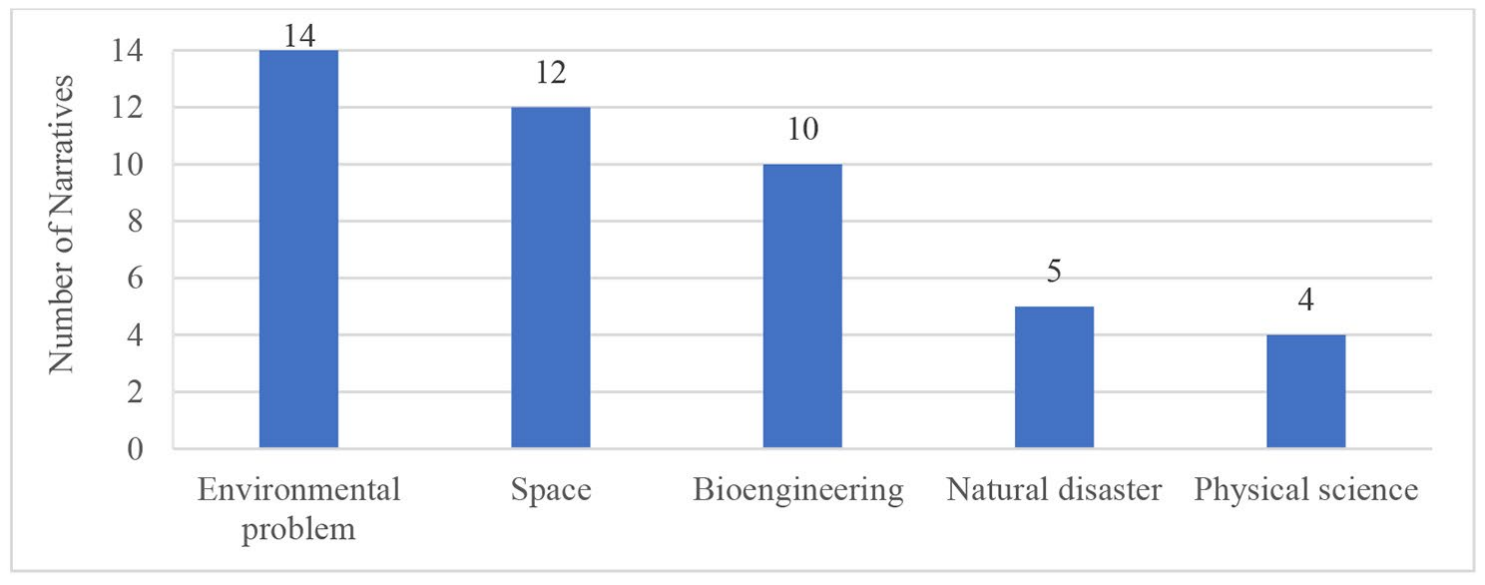
Number of Narratives that Contain Science Topics *Note*. Each narrative may include multiple topics, so the total does not add up to 35

The analysis showed that the narratives included diverse science topics (Fig. 2), despite the fact that our program was designed to focus on the theme of environment and human health. The most popular science topic in these narratives was environmental problems caused by human activities – 14 narratives covered issues such as pollution, global warming, endangered species, sea-level rise, deforestation, and destruction of the ozone layer. The next most popular topic was about space exploration, including themes such as alien life, wormholes, discovery on other planets, asteroid crashing, and space travel. Bioengineering (i.e., cloning, bionics creation, new species discovery, mutation of animals, and gene variation) was also popular. Natural disasters (i.e., hurricanes, tsunamis, and tornados) and physical sciences (i.e., nuclear explosions, radiation, energy conversion, and chemical elements) were also presented but not as popular.

Moreover, most of the narratives involved more than one science topic, and different methods were employed to connect these topics. Some narratives presented a nesting structure of science topics – one science topic was presented as the major theme, and the other topics were introduced through characters, events, obstacles, or conflicts/climax. For example, in S1 (in the following, we use ‘S#’ to represent each narrative, see Appendix B for a list), the major science topic was Earth’s pollution, and the topic of cloning was introduced through one of the characters in the narrative (a cloned sheep). In another example (S4), the primary science topic was about an endangered species on Earth (e.g., turtles), and the topic of extraterrestrial life was integrated through the central event in the narrative (aliens coming to Earth to save the turtles). In S28, the original mission was to explore a new planet, but this mission was hindered by an unexpected wormhole; so, the protagonists ended up travelling to the future through the wormhole and found different forms of trash spread everywhere on Earth.

Another popular cross-topic connection structure was the sequential occurrence of science topics, often linked by causal relationships. For example, in S31, asteroid crashing, the first science topic presented in the narrative, leads to the occurrence of another one – the species extinction on Earth. In S17, the original purpose of a sea exploration mission was to discover new species, but the explorers later found a canister with rare chemical elements.

### Theme 2: integrating Science through Narrative techniques

Along with the various ways in which the different science topics were connected, the ways in which the science contents were integrated into the narratives also varied a great deal. Three different narrative techniques were discussed below.

*Character dialogue.* Character dialogue was one of the most prevalent ways to incorporate science into these narratives. For example, in S23, Alice (the protagonist) asked, “So I understand that Elsa is evil, but I still don’t understand how she can heat up Earth to such a horrible extent.” This kind of question by a character created a catalyst for immediately expanding science information in the narrative. In this case, Senora Hearts (another character) explained, “Elsa is causing global warming with her heat powers. Global warming is a gradual increase in the overall temperature of Wonderland’s atmosphere generally attributed to the greenhouse effect, caused by increased levels of carbon dioxide, chlorofluorocarbons, and pollutants. Wow, what a mouthful.” The dialogue between Alice and Senora Hearts elucidated the definition and cause of global warming in the fictional world in their narrative—the Wonderland. Even though the definition of global warming might be copied online directly, students were making an attempt to embed science contents into a specific narrative context through character dialogues.

*Action revelation.* Another way of science integration was through describing character actions. Most of these actions were from students’ daily experiences and life events, such as surfing the Internet, reading newspaper, or watching TV. For instance, in S4, the leader of the alien turtles found out information about endangered turtles on Earth by reading the latest news online. In S19, for another example, the science information that a mysterious green creature was fond of pop music was incorporated when a character was reading a newspaper.

*Explaining events or plots.* Naturally, science information was usually inserted to explain some key event(s) presented in the narratives. These events could be proximal or distal with respect to the science information. For example, in S17, the science sentence: “Their research said that a Goliath Grouper could eat a small shark. So at least the explorers were protected by the Grouper”, was inserted right after the sentence, “The explorers were happy to see the giant grouper, and it didn’t attack the explorers.”. In this example, the science information served to explain why the explorers were happy to encounter the grouper. In another example, in the middle part of S26, where the science information regarding the cause of the tsunami was inserted, it was connected to the beginning event that a tsunami had happened. In S9, the science sentence that described speeds of hurricanes and tornados was mainly used to provide a context for the later event in which the characters needed to calculate how much time they had left to make up their plan to stop them.

### Theme 3: proposing alternative solutions for Science-related problems

Echoed with the diversity of science topics, a wide array of science-related problems, such as global warming, extinction of species, and pollution, was presented in students’ narratives. Twenty-six narratives contained at least one science-related problem, and 20 of them provided specific solutions for their problems. In the following, three interesting subthemes regarding the nature of these solutions were described.

*Realistic vs. unrealistic.* The solutions proposed in the narratives differed with regard to feasibility on the basis of current knowledge and technology, with the understanding that it was often hard to draw a line between realistic and unrealistic solutions as today’s sci-fi might become tomorrow’s reality.

There were eight narratives offering more or less realistic and pragmatic solutions, including inventing a device to produce breathable air (S10, S31), designing an oxygen tank (S11), and manually picking up the trash to save the polluted Earth (S28). Given the age group of the participants, solutions in other narratives (*n* = 9) tended to be unrealistic, or purely fantasy in nature. These included leaving Earth behind and living on another planet when the Earth was contaminated (S1), moving to an underwater city after the crumble of Earth (S7), relying on superpowers and superheroes to stop a tsunami (S9), and using a treatment serum to heal zombies (S15). Some narratives included both realistic and unrealistic elements. In S23, two alternative solutions were suggested to stop global warming in the wonderland where it was caused by Elsa’s heat powers and Ariel’s bubble power (both Disney characters) that could trap the heat inside the atmosphere. Plan A was to stop Ariel’s bubble power for releasing the heat, which was seen as a fictional one, and Plan B was to pick up the trash to save their living world.

*Optimistic vs. pessimistic.* Employing science and advanced technology was a common theme in problem solving through the narratives. Students’ solutions reflected the differentiated attitudes and perception regarding humankind in facing and solving the portrayed problems.

A total of 11 narratives clearly presented an optimistic attitude towards science or technology, and their solutions successfully resolved the problem at the end. In S10, the world was falling apart because of global warming and air pollution. The main characters, a chemist and a marine biologist, created a device that purified the greenhouse gas and saved Earth.

In comparison, the same number of narratives (*n* = 11) showed pessimistic attitudes towards science and technology. In many cases, a scientist’s experiment led to the apocalypse. For example, in S30, four scientists created a machine to produce water, but the test process went wrong. The malfunctional machine generated heat to melt all the ice caps on Earth, and sadly, no solution was offered in the narrative.

*Group collaboration vs. individual development.* A total of 19 narratives provided explicit information regarding whether the problem stated in the narratives was resolved by groups or individuals. It was interesting to see that the majority (*n* = 17) of sci-fi narratives portrayed the problem-solving processes as collaborative, whereas only two narratives portrayed individual-based solutions for tackling real problems. Part of this could be related to the instructional strategy in the program: Students had to complete their projects in small teams.

Collaboration occurred in different stages, across areas of expertise (i.e., chemist, marine biologist, geographer, or peoples with different superpowers), races and countries, different species (i.e., humankind, aliens, and animals), and even opposing sides. For example, S9 described an ongoing collaboration and work distribution among four characters with different superpowers for stopping a “turricane” (a hypothetical natural disaster that combines hurricane and tornado). Additionally, some narratives also presented the obstacles of successful and effective collaboration, such as the betrayal of allies, greediness and selfishness, or incapable leadership.

### Theme 4: integrating Science through Multimodal Design

While some narratives were presented in a single mode (six in written texts and two in comics only), the majority of students’ narratives (*n* = 27) were multimodal in nature. They presented a combination of a variety of multimodal elements beyond texts, including comics, diagrams, graphs, videos, background music, 3D simulation, audios, and drawings. When examining the science content contained in these multimodal elements (i.e., the science dimension) and considering how these elements were connected with the rest of the narratives (i.e., the integration dimension), the following observations were derived.

**Fig. 3:**
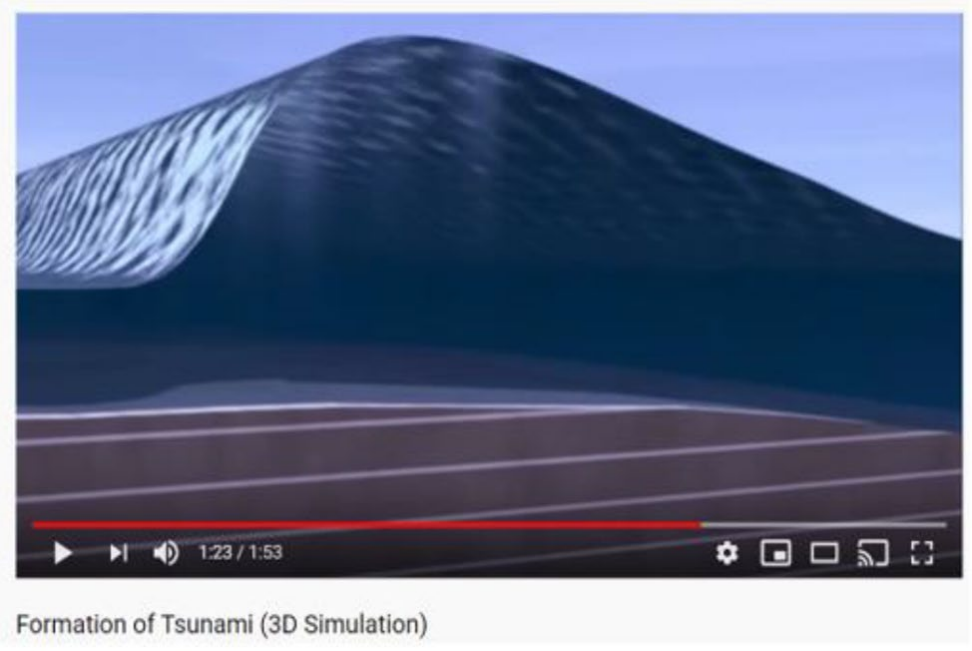
3D Simulation Video from YouTube in S26

The multimodal elements were used to introduce, explain, and further extend the science information in the narratives. Communicating with multiple modes offered students extended options for crafting their narratives. The most popular form of online multimodality resources was science videos or 3D animations. For example, in S26, there was a sentence about the cause of tsunamis: “The Squad knew that Earth’s tsunamis were caused by seismic activity and the moving of plates, even the smallest movements cause a huge wave”. As the sentence did not explain why the small movement of plates would lead to a huge wave (tsunami), the narrative included an embedded 3D simulation (Fig. 3) that showed visually how the slight movement of tectonic plates first caused small waves, then while approaching the land, these waves continued to grow with much increased amplitude and eventually formed a tsunami.

**Fig. 4:**
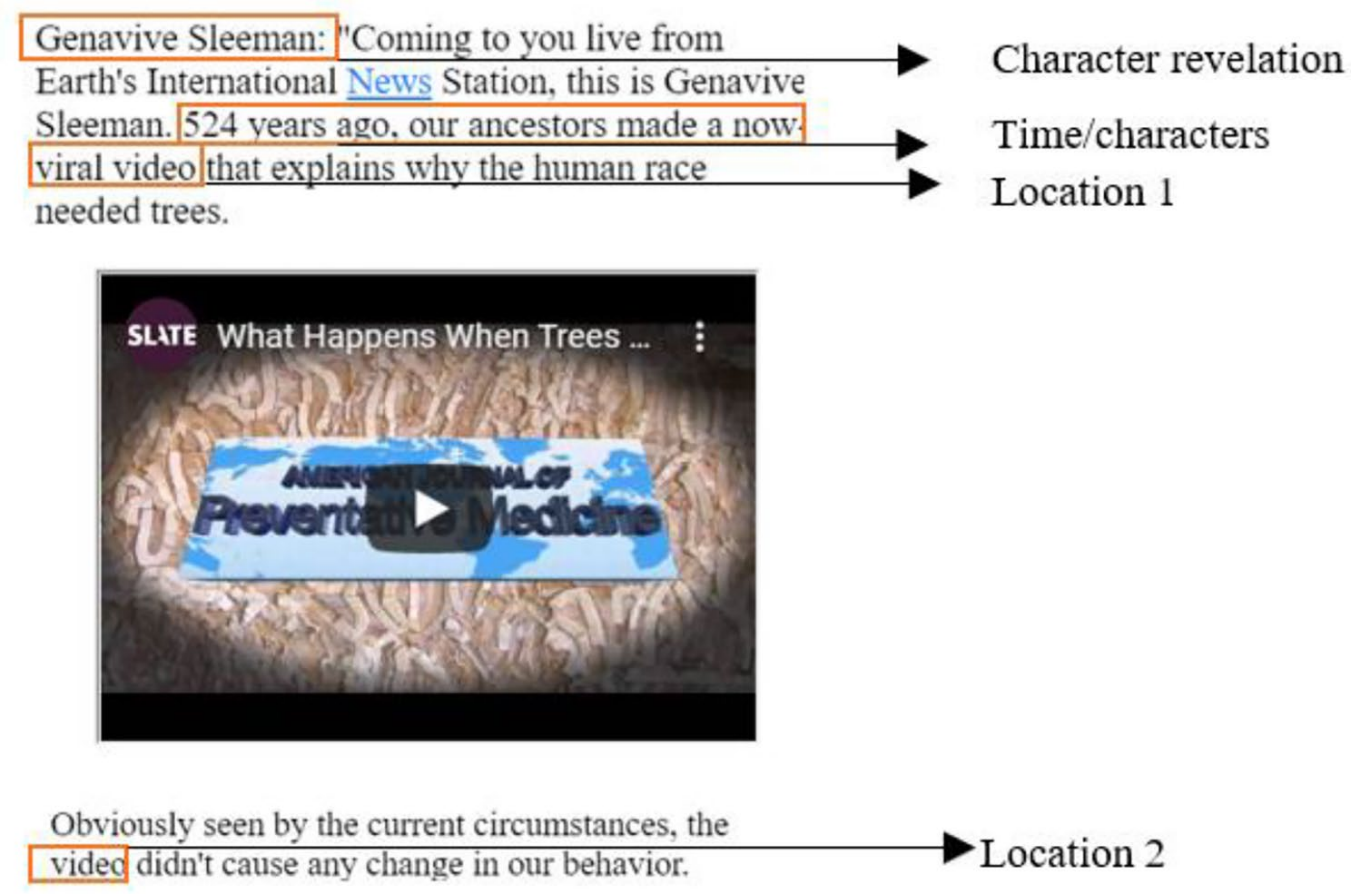
A Video Screenshot on Deforestation in S18

Considering integration, many of these multimodal elements were carefully selected and deliberately placed to make them an inherent part of the narratives. S18 presented a good example that showed how a YouTube video on deforestation was well integrated into the narrative (Fig. 4). The video was part of a broadcast by the main character, Genavive Sleeman. According to the broadcasting, the video was created 524 years ago to increase people’s awareness about the significance of trees. The text right after the video provided a hint that a twist of the plot was coming (the mechanism introduced in the video did not work). In this way, the video was effectively woven into the narrative and connected to multiple aspects of the narrative (characters, time, and plot).

**Fig. 5:**
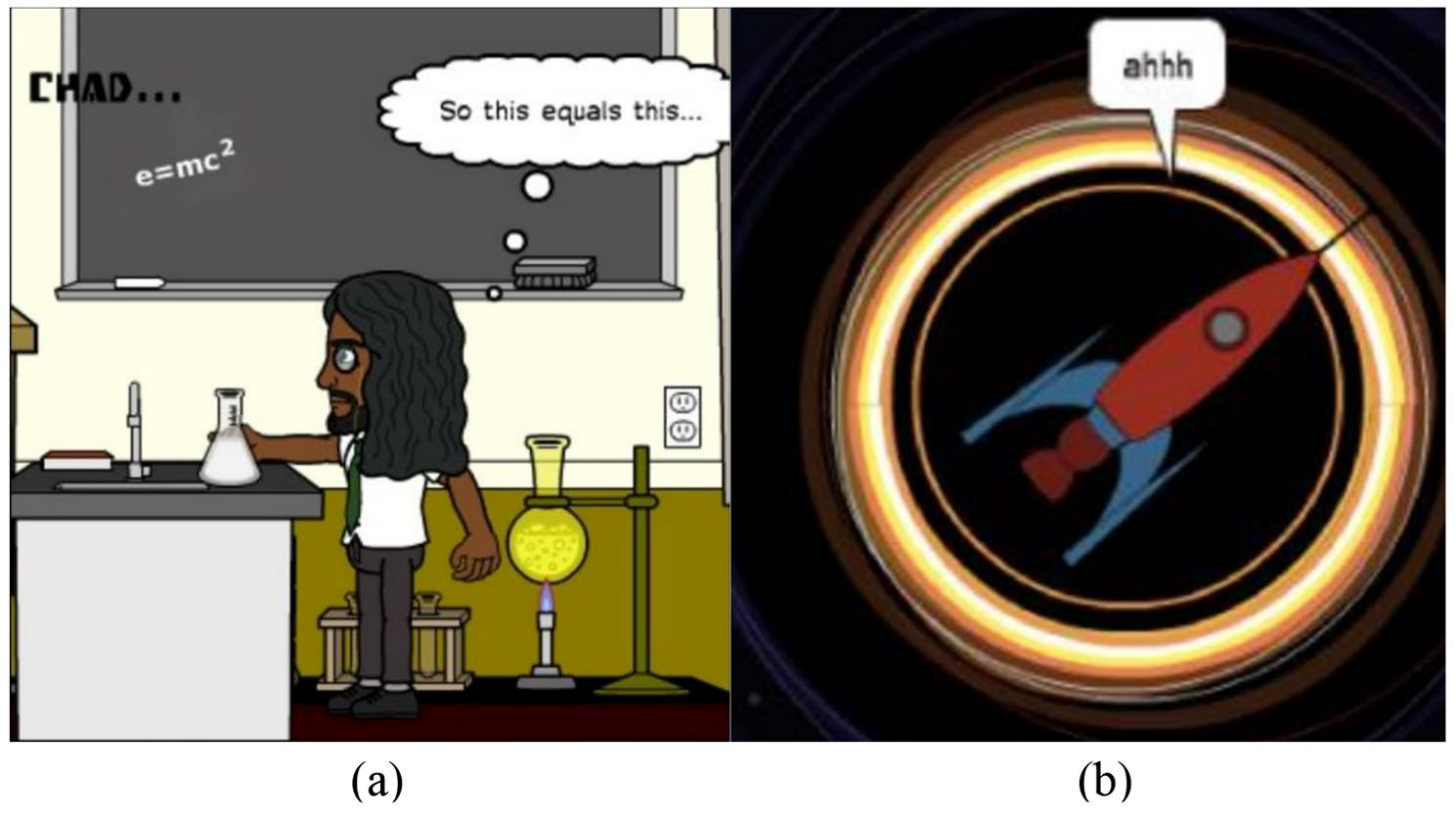
Portrayal of a Scientist Working in a Lab in S15 (a) and a Spaceship Traveling through a Wormhole in S28 (b)

While the aforementioned examples showed how students used and inserted existing multimodal resources from the Internet into their narratives, there were also abundant examples where students themselves designed and created science-relevant multimodal elements. The most common type of multimodal elements that students created were comics. For instance, in S26, the cause of global warming and air pollution was first explained in written texts, then represented in the form of a comic. In S15, a scientist was portrayed as a Black male with long hair in a typical science lab setting with the famous equation *e = mc*^2^. In S28, a wormhole through which a spaceship was traveling was portrayed vividly and colorfully (Fig. 5).

**Fig. 6:**
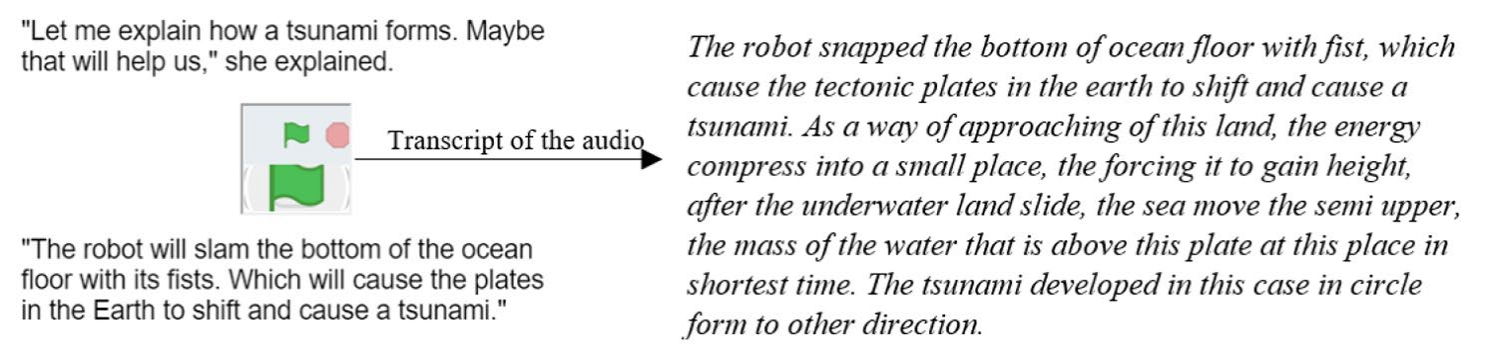
The Use of Self-recorded Audio in S29

Audio narration was another type of multimodal representations that students integrated into their narratives. In S29, students composed their own audio clip made in Scratch (Fig. 6) to record a precise verbal explanation of how a tsunami was formed. Audio narration allowed students to use their own voices to talk about science. In this case, the audio file was inserted right after “she explained” in the texts, making the audio element a natural part of the narrative.

Despite that in many cases the multimodal elements and the corresponding texts both contained the same science information, reconfiguring across different modes went beyond simple replication. The process involved a series of meaning-making translations and transformations, such as rewording (“warm up” to “heat up”, “trap the heat inside the atmosphere” to “trap the heat with your gas bubbles” in S26) and rethinking the meaning of science through visualization (e.g., mixing stereotype and non-stereotype elements in one comic panel).

## Discussion

The analytical framework used in this study is a result of the need to assess students’ integration of science knowledge in multimodal compositions. Research has stressed the importance of having clear rubrics in examining students’ generated compositions (Crusan, 2010, 2015; Glasswell et al., 2001; Odell & Katz, 2009). However, most assessments of students’ multimodal compositions have not been adapted for evaluating science components within narratives. The analytical framework developed in this study offers a practical way to evaluate student-generated sci-fi narratives. Extending to broad science-related topics and problems beyond narrow concepts, we see the rich and diverse scientific content that students incorporated in their narratives. More importantly, this work is seen as initiating an important approach to analyzing students’ application of science, that is, integrating science with narrative characters, settings, events, and plots, and using science as a foundation to help characters deal with various real-world problems. This implies that students’ learning of science should not be separated from how science can be useful in problem situations.

This study shows that the quality of the science dimension (including the concept/phenomena and problem/solution aspects) on average was relatively low. This is not unexpected as the essence of students’ final products is a fiction. On the other hand, the sci-fi narratives together demonstrate quite sophisticated mechanisms that the students used to integrate science in their narratives. First, these narratives included diverse science topics and built cross-topic connections; second, they showcased narrative techniques to purposefully incorporate science into their narratives; third, they presented a wide range of creative responses or solutions to popular socio-scientific problems; and fourth, they incorporated and mixed multimodal elements to enhance science integration. Echoing the literature (i.e., McDermott & Hand, 2010; Norris et al., 2005; Yore et al., 2003), this study demonstrates that narratives have a great potential in bringing in students’ rich personal experiences and their own vocabularies (for instance, in one narrative, students coined the term “turricane” to denote a hypothetical natural disaster that was a combination of hurricane and tornado). This is crucial for young learners to enter the STEM fields as they are able to bring with them prior knowledge, existing beliefs, and individual perspectives (e.g., Rousell & Cutter-Mackenzie-Knowles, 2019). Furthermore, consistent with previous research (Kosky, 2014; Morrison & Chisin, 2017; Rousell, 2017; Vrasidas et al., 2015), this study also demonstrates that the sci-fi genre is suitable for engaging students in inquiring, incorporating, and connecting various broad science topics with their own interests.

Additionally, studying the features of students’ final products gives us hints on what benefits this kind of curriculum could bring to students. Taking the social semiotic perspective (Kress, 2003; Kress et al., 2001), the unique ways of incorporating multimodal elements in each of their narratives reflect students’ personal engagement and exploration in meaning-making and self-expression. The observations during the project implementation also indicate that this process is often highly engaging for the students in this study. Moreover, considering their age group, the diverse ways of translating and transforming multimodal semiotic resources and representations (Ainsworth, 1999, 2006; Shen & Confrey, 2007; Prain & Waldrip, 2006) also illustrate the high level of creativity and authenticity of students’ products. Students attempt to carefully weave the multimodal components in their narratives and associate these components with science and other narrative elements, in order to make their narratives more coherent. This is an important learning experience given that nowadays they are living in a multimodal world, and “the job of producing coherence, a responsibility traditionally borne by the author or lecturer, has now devolved on the reader or viewer” (Scardamalia & Bereiter, 2014, p.402). Promoting coherence is also imperative in learning complex science topics, such as socio-scientific issues. For example, consider the information in diverse modalities we need to consume and make sense in dealing with the ongoing Covid-19 pandemic.

Despite the encouraging findings, this study has limitations. First, this study only focused on analyzing students’ final products through proposing and applying an analytical framework, but the framework itself needs further validation. It is also important to note that very little is known about how students apply and integrate science into their multimodal sci-fi narratives; in other words, this study is exploratory in nature. It is necessary for us to develop a framework for helping better understand students’ final products. We believe the framework itself is a contribution to the field, and it may serve as a starting example for those who are interested in similar topics. In the future, such as analytical framework needs further research and validation. Second, related to the first point, in this study, we did not examine the processes of how students employed different strategies to produce those artifacts, which was partially explored in our other studies (Smith et al., 2019). Much more future research on examining students’ learning processes and understanding how these processes contribute to different products is still needed. Third, many narratives in the sample lacked depth in explaining science concepts. This might be partly due to the tension between fictional narratives and scientific coherence (Matuk et al., 2019). On the one hand, the speculative aspect made it more attractive and engaging for students. On the other hand, these imaginative elements could steer students away from deepening their understanding and explanations of the science concepts. Future research is needed to further examine the balance between science content and fictional elements in sci-fi narratives. A relevant issue regarding inserting multimodal science elements was that it was not known how much science students have learned from these resources. Future research about examining how students use these resources to facilitate their science learning is recommended.

## Conclusion and implications

In this study, student-generated multimodal sci-fi narratives were examined via a two-dimensional analytical framework that was developed for capturing how students integrate science elements. The results revealed notable characteristics and patterns of student-generated multimodal narratives and showed multilayered mechanisms that students used to integrate science in their narratives. These mechanisms involved interdisciplinary learning experiences across science (building cross-topic connections among diverse science topics), literacy (using a wide range of narrative techniques to enhance the narratives’ coherence), and technology (designing multimodal components through digital tools). The results also demonstrate that multimodal sci-fi narrative composition may open spaces for adolescents to speculate the radical changes and consequences of human actions, as well as to propose solutions in response to the distressing environmental problems and fast-changing technologies. The combination of digital tools and sci-fi writing allow students to go beyond what is “possible” now and to imagine the future.

The results of this study suggest that the use of multimodal narratives, especially in the genre of fiction, can stimulate students’ creativity and imagination to respond to the natural environmental and social problems that all humans have faced. Besides, the form of narrative is convenient for students to extract story materials from their own real-life experiences (i.e., reading newspapers or watching TV). Thus, the composition of science fictional narratives is seen as an effective method that can be extended to different cultures and contexts.

Pedagogically, student examples of digital sci-fi narratives can inform us how to better help them integrate science and literacy. The guidance of the iKOS platform, ConceptMap, and other various multimodal composition tools can serve as valuable venues for students to develop a blueprint for their digital story design. Engagements in and discussion about hands-on science activities and big science concepts can inspire students’ creations and assist them in brainstorming possible core science elements and layouts of their digital narratives. In addition, exposures to good narrative examples and writing structures may have the potential to help students learn different ways of integrating different modes for a better narrative. However, locating free science and multimodal resources from the Internet is now a common act due to easy accessibility. More guidance is needed to provide students with the selection and integration of extant online recourses.

## Electronic supplementary material

Below is the link to the electronic supplementary material.

Supplementary Material 1

Supplementary Material 2

## Data Availability

Not applicable.
