# Supplementary material for: Creating Digital Sci-Fi Narratives through Multimodal Composing to Promote Adolescent Students’ STEM Education
Source: Discip Interdscip Sci Educ Res. 2023 May 12;5(1):7. doi: 10.1186/s43031-023-00072-7 (PMC10176294; doi:10.1186/s43031-023-00072-7)
Supplement: Supplementary file 1 — Supplementary Material 1 [file 43031_2023_72_MOESM1_ESM.docx]

Appendix A

*Rubrics for evaluating multimodal sci-fi narratives and examples*

| Dimensions | Aspects | Levels/scales | Explanation | Examples |
| --- | --- | --- | --- | --- |
| Science | Concepts/  phenomena | Level0: 0 point | No science concepts or phenomena were mentioned |  |
|  |  | Level1: 1 point | The science concept/phenomenon is only briefly described in text and other modalities, and none or only one of the three criteria is barely satisfied. | S5: A comic containing two science sentences  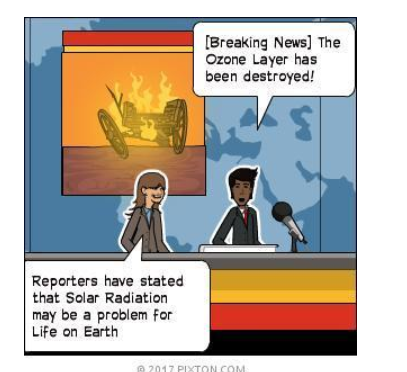  In these two science sentences, two science concepts — “ozone layer” and “solar radiation” were mentioned without definitions or further depict. |
|  |  | Level2: 2 point | The science concept/phenomenon is described in text and other modalities, and one or two of the criteria have major weaknesses. | S9: "*Ok. Guys look at this caterpillar" said Alex.*  *"Do you know that in a few weeks it will turn into a cocoon then a beautiful butterfly? "said Jacob." Yeah, but sadly since we live on the moon there getting extinct."*  Students used three accurate terminologies (caterpillar, cocoon, and butterfly) to describe the life cycle of butterflies and just provided the information of time (i.e., a few week), while no more other specifies were given. |
|  |  | Level3: 3 point | The science concept/phenomenon is accurately and clearly described in text and other modalities and has sufficient details. | S18:  Sentence1: “*Because of the ignorance that deforestation was taking place, many people didn't know what a huge threat they were to the environment.*”  Sentence2:  “*Ever since, the rate the human race produced carbon dioxide through gasoline and fossil fuels, the carbon in the stratosphere increased so much that the remaining trees couldn't produce enough clean oxygen to sustain life*”  YouTube science video link: what happens when trees disappear (<https://www.youtube.com/watch?v=gHVr7FWAEp0&feature=emb_logo&ab_channel=Slate>)  The first sentence pointed out that the deforestation would cause a huge environmental threat to human. By reading to the second sentence, readers could know the environmental threat was no enough clean oxygen on Earth. Also, the second sentence presented a clearer relationship between the increase of carbon dioxide, deforestation, and reduce of breathable air.  The science YouTube video provided sufficient details about the consequences of deforestation to environment and people life. |
|  | Problem/ solutions | Level0: 0 point | No science-related problems were included |  |
|  |  | Level1: 1 point | The problems were briefly mentioned in text or other modalities. No solutions were offered or the solutions were unrelated to the problem | S1: Earth and ocean pollutions were solely brought up without an explanation of what caused the pollutions. Further, no solutions were provided. Protagonists abandoned the Earth and moved to another planet called Phobos. |
|  |  | Level2: 2 point | Analyses about problems were provided, but solutions had major flaws either in plausibility or suitability | S18: The problem in the narrative was that there was no clean oxygen for people’s life. The cause of this problem was the deforestation over years, as a result, over amount of carbon dioxide were discharged into stratosphere and the remaining trees cannot produce enough clean oxygen, at the same time, the machine created by Dr. Fezz, which was used to produce oxygen, had suddenly malfunctioned. The solution for this problem was four main characters would travel to space to fix the machine. The narrative gave a good narrative about why the solution was hard to implement because of different purposes of characters and noncooperation between them. But there was no more information about how to fix the machine or how to directly solve the problem. |
|  |  | Level3: 3 point | Complete analyses about problems, plausible and suitable solutions were provided | S10: The text and comic both provided explanation about the cause of global warming. The text indicated that it was because of the war and factories that caused the global warming. The comic provided more specific explanation that the U. S had many factories to keep up with the demand for war supplies, and this ended up with putting lots of pollution in the air. The narrative mainly discussed about the solutions for air problems from chapter 4 to chapter 6. The remaining human needed to invent a device to turn CO2 into O2. First, they found chemicals in a vault that could be used to create vaccines. Second, they made a machine to scan and purify the greenhouse gasses. Last, they run an experiment to test on the wildlife and proved the air had no side effects. The solutions were implemented step by step and successfully solved the air problem. |
| Integration | Integration between science and narrative | Level0: 0 point | Does not meet any of the three criteria |  |
|  |  | Level1: 1 point | Meet one of the three criteria | S4: The main science sentence that called people’s attention to endangered turtles was inserted outside of narrative and no narrative techniques were used to integrate this sentence into story plot. It was only related to the previous event that Blair (character) was making some plans to save turtles |
|  |  | Level2: 2 point | Meet two of the three criteria | S27: The main science sentence “*That day, Jenny and Mrs. Smith spoke about the Solar Eclipse and the chances of seeing other planets with the naked eye*” was inserted in the right place where Jenny was recalling the conversation with Mrs. Smith, thus, it was revealed through the narrative characters. However, the science was not related to the later event that Jenny had an adventure to an alien planet. |
|  |  | Level3: 3 point | Meet all of the three criteria | S9: “*Ok so according to my analysis, the wind speed of the hurricane is 560 miles per hour, and the tornados speed is 201, the turricane has a total speed of 761 miles per hour, says Liam*”.  This science sentence was revealed through character dialogues and closely related to later event that all the characters were making a plan to stop turricane based on Liam’s calculation. |
|  | Integration between problem and narrative | Level0: 0 point | No integration was presented |  |
|  |  | Level1: 1 point | The problem was only mentioned in one of three scenes and the other two scenes were totally unrelated or weakly related to the problem | S5: The environmental problem in S5 referred to the destruction of ozone layer. The beginning and middle of the narrative is mainly about Isa’s discovery of her own diary and her job interview. No specific aspects or hints of the environment problem were described. |
|  |  | Level2: 2 point | The problem(s) were mentioned in two scenes of narrative | S23: Global warming and air pollution in the wonderland were the main science-related problem in S23, which was first presented at the middle of narrative when Alice was accidently stepped into the wonderland. The narrative went to the climax when Alice found Senora Hearts and Mad Hatter pretended that Alice was the only one to save wonderland from global warming. Indeed, they conspired to set up a trap for destroying Alice. While there is no clue of such environmental problems in the first scene when describing Alice’ daily life. |
|  |  | Level3: 3 point | The narrative was clearly unfolded around one or two main problems from the beginning to the end. Problem (s) played a significant role in the three scenes of narrative | S26: the narrative is clearly unfolded around the main problem “tsunami”. In the beginning, the writers directly point out the problem, “*there is a huge wave coming for us*”, “*oh, no it is a tsunami*!!!”. Then the narrative described the consequence caused by tsunami. The next scene is unfolded around saving people and coming out a plan to stop tsunami. The last part mainly talked about the implementing the plan of using sponge to sack up waters and stop tsunami. It is very clear that the problem led story event development and finally reached to the resolution. |
