# Supplementary material for: Creating Digital Sci-Fi Narratives through Multimodal Composing to Promote Adolescent Students’ STEM Education
Source: Discip Interdscip Sci Educ Res. 2023 May 12;5(1):7. doi: 10.1186/s43031-023-00072-7 (PMC10176294; doi:10.1186/s43031-023-00072-7)
Supplement: Supplementary file 2 — Supplementary Material 2 [file 43031_2023_72_MOESM2_ESM.docx]

Appendix B

*Descriptive Information for 35 Multimodal sci-fi Narratives*

| Sci-fis | Title | Main story line | Science topics/concepts | Multimodality |
| --- | --- | --- | --- | --- |
| S1 | 3002 | A human and a clone sheep travelled to the Earth and found the Earth was so polluted. | Earth pollution and clone | Text, music, and comics |
| S2 | A leap into the future | Four students were planning to create bionic parts of one of them but something went wrong. | Bionic creation | Text and comics |
| S3 | A new star | A wondering adventure on an alien planet called the Haluki star. | Space travel | Text and animations |
| S4 | A turtle tale | The alien turtles on a remote planet traveled to the Earth for saving the endangered turtles. | Endangered creatures, and space travel | Text, comics, music, and images |
| S5 | Breaking news | A marine biologist discovered a CD that described the future disaster of destroyed ozone layers in 2030. | Destroying of ozone layer | Text and comics |
| S6 | Captain Atomicon | A superhero who gained the power from nuclear explosion fought against a psycho and his cyborg army. | Genovariation and nuclear explosion | Text only |
| S7 | Essent Koeln | The citizens of Tallin had to conquer an underwater futuristic city-Essent Koelen, for keeping their civilization when Tallin was beginning to crumble due to pollution and erosion. | Earth pollution and recycling | Text and animations |
| S8 | Galactic war one | Two young people took a spaceship and discovered a new planet. | No science | Comics only |
| S9 | How Could This Happen to the Moon | A bunch of people with superpowers stopped the devastating turricane and prepared for the next mission to save the extinctive butterflies on a moon. | Extinction of creation and hurricane | Text, diagram, and music |
| S10 | How it happens | After a nuclear war, scientists and government created a device that produced respirable air for saving people from global warming . | Global warming, and oxygen reproduce | Videos, text, music, comics, and games |
| S11 | Mark and The Aliens | Mark escaped to a planet called Tentaclus, but got caught in a war between the King and his rebels. | Space travel and rocket propulsion | Text, amination, and music |
| S12 | Overwatch | Overwatch combatted the Blackwatch, an evil organization that was planning to destroy the world by melting icebergs. | Ice burg melting and sea level rising | Text, comics, and music |
| S13 | Pokemon, the story | A mad scientist created a machine to turn the world into a dark type. | Alien | Text, music, comics, and image |
| S14 | Project Cor 3 | Alien attacked the Earth for plundering the cor3, a type of energy that was useful to cool down their overheating planet. | Overheating and Alien | Text only |
| S15 | Research Gone Wrong!!! | Few people struggled for survival in a world of zombie raging. | Genovariation and water discovery on the Mars | Text, comics, and images |
| S16 | Strangest thing | Conor fell into an alternative world by mistake, then his three friends set out on the hard road to rescue him. | Forest pollution | Text and images |
| S17 | The Aqua Doods | Two sea explorers went out to the ocean to explore the Mariana Trench for seeking new species of sea life. | New special discovering, and chemical elements | Text, images, animations, comics, music, and figures |
| S18 | the big surprise | A machine that could transfer carbon dioxide into breathable oxygen has suddenly malfunctioned and survivors went toward space center to fix it. | Global warming, deforestation, and photosynthesis | Text, vides, and image |
| S19 | The eclipse ostriches | Four characters worked out on identifying an alien creature from space during eclipses. | New species discovering | Text and comics |
| S20 | The Great Sand Demon | Several people were stuck in the middle of dessert and one of them was bit by a weird animal then started acting weird and crazy. | Mutation of animal | Text, comics, and music |
| S21 | The life of Larry | Larry could not go to his school because of flooding and he was caught in the daydream for five years | No science | Text and comics |
| S22 | The new planet | Three girls traveled to a planet and settled down. | Space travel and exploration | Comics only |
| S23 | The Nightmare in Wonderland | In the wonderland, Alice aided Mad Hatter, and Senora Hearts to stop global warming caused by the evil queen, while it turned out they collectively set a trap for killing Alice. | Global warming and air pollution | Text, comics, music, and videos |
| S24 | The pandaries | Two teenagers took a rocket to space for discovering new planets. | Space exploration | Text, comics, animation, videos, and music |
| S25 | The revolution | A secret base started a revolution against the corrupt leadership who has demolished Chicago. | No science | Text only |
| S26 | The Squad | Five supermen rescued a whole city after the tsunami happened. | Tsunami | Text, comics, and videos |
| S27 | The story in the space | Jenny, a middle school student, had a daydream in an alien planet. | Space exploration and alien | Text only |
| S28 | The wormhole to the future | A half alien, half human traveled to the Earth future through the wormhole, while discovered the Earth was so polluted. | Earth pollution, new planet exploring, wormhole, and alien | Text and comics |
| S29 | Tsunami Terror | Kai, an evil alpaca, was planning to destroy the world through starting a tsunami and the other three superheroes were attempting to stop this crazy plan. | Tsunami | Text, image, comics, music, and audio |
| S30 | Water: The Beginning and the End | Four scientists created a machine for producing water, but unexpectedly caused a tsunami and wiped out the whole human. | Sea level rising and tsunami | Text, image, comics, music, and figures |
| S31 | What would happen if the world stopped spinning? | After the asteroid crashing, the Earth stopped spinning, animal species died out, and only three people survived. | Species extinction and asteroid crashing | Text, animation, comics, and images |
| S32 | Journey to Empire of the Reptilian King | Two characters travel through a wormhole to rescue a partner only to get caught in a war between the Reptilian King and his rebels. | Wormhole | Text only |
| S33 | Project 115 | A super soldier sent to an underground lab to be cloned is on the run and causing damage | Cloning | Text only |
| S34 | Teleportation | A teleportation technology created by moon colonies was used to overthrow the earth’s colonialism | Alien, energy converting, and nuclear explosion, and radiation | Text only |
| S35 | Chickens Strike Back | Scientific chickens made a machine to generate nature disasters (i.e., hurricane or tornado) for revolting that human took chickens as food. | Tornado | Text, comics, and figures |
